# The value of the simplified RAMRIS-5 in early RA patients under methotrexate therapy using high-field MRI

**DOI:** 10.1186/s13075-018-1789-3

**Published:** 2019-01-14

**Authors:** Miriam Frenken, Christoph Schleich, Ralph Brinks, Daniel Benjamin Abrar, Christine Goertz, Matthias Schneider, Benedikt Ostendorf, Philipp Sewerin

**Affiliations:** 1 Department for Diagnostic and Interventional Radiology, UKD, Heinrich-Heine University Düsseldorf, Moorenstrasse 5, 40225 Düsseldorf, Germany; 2 Department and Hiller Research Unit for Rheumatology, UKD, Heinrich-Heine University Düsseldorf, Moorenstrasse 5, 40225 Düsseldorf, Germany

**Keywords:** High-field magnetic resonance imaging, Early rheumatoid arthritis, Prediction, RAMRIS-5, RAMRIS

## Abstract

**Background:**

The aim of the study was to evaluate a simplified version of the Rheumatoid Arthritis Magnetic Resonance Imaging Score (RAMRIS) for five joints of the hand (RAMRIS-5) in patients with early rheumatoid arthritis (RA) before and after the initiation of methotrexate (MTX) therapy using high-resolution, 3-T magnetic resonance imaging (MRI).

**Methods:**

Twenty-eight patients with a seropositive, early RA (disease duration of less than 6 months (range 2–23 weeks)) according to 2010 American College of Rheumatology/European League Against Rheumatism (ACR/EULAR) criteria (mean age 56.8 years, range 39–74) were prospectively assessed with a baseline investigation including clinical assessment (disease activity score of 28 joints (DAS-28) and C-reactive protein (CRP)) and 3-T MRI of the clinically dominant hand. Follow-up visits were performed 3 and 6 months after initiation of a MTX therapy at baseline. MRI scans were analyzed in accordance with RAMRIS and the simplified RAMRIS-5.

**Results:**

DAS-28 and CRP decreased significantly after initiation of MTX therapy. Even though erosion scores increased over time, RAMRIS and RAMRIS-5 also decreased significantly after the start of therapy. There was a strong correlation between the total RAMRIS-5 and RAMRIS at baseline (*r* = 0.838; *P* <0.001) and follow-up (3 months: *r* = 0.876; *P* <0.001; 6 months: *r* = 0.897; *P* <0.001). In the short term (3-month follow-up), RAMRIS and RAMRIS-5 demonstrated similar ability to detect changes for all subgroups (bone edema, erosion, and synovitis). In the long-term comparison (6-month follow-up), RAMRIS-5 also showed similar effectiveness when detecting changes in bone edema and erosion compared with RAMRIS. Deviations occurred regarding only synovitis, where change was slightly higher in RAMRIS-5: SRM (RAMRIS) = 0.07 ± 0.14; SRM (RAMRIS-5) = 0.34 ± 0.06.

**Conclusions:**

Three-Tesla MRI-based RAMRIS-5 is a simplified and resource-saving RAMRIS score which compares favorably with the RAMRIS when detecting changes in early RA. Even though there is a slight abbreviation between RAMRIS-5 and the original score regarding the change of synovitis, it may be used for diagnosis and therapy monitoring in follow-up evaluations.

## Background

Rheumatoid arthritis (RA) is the most common inflammatory joint disease. Early diagnosis and treatment significantly improve the long-term outcome [1, 2]. Delayed treatment leads to chronic synovitis, joint destruction, pain, loss of function, and reduced quality of life [1–3]. Many studies have shown that early and rigorous treatment significantly reduces the impact of chronic inflammation and prevents radiological progression in a large proportion of patients [3, 4]. In the early stage of the disease, there is a “window of opportunity”, which starts to close between the third and fourth month after symptom onset where patients have more benefits from active treatment with conventional synthetic disease-modifying anti-rheumatic drugs (csDMARDs) than in the later course of the disease. This is because irreversible joint destruction occurs more often in the later stages of the disease [5]. Therefore, the current treat-to-target approach demands therapy with csDMARDs at an interval no more than 3 months after symptom onset. Thus, early diagnosis is crucial to allow early treatment and prevent irreversible joint destruction [6]. In early stages, RA often shows non-typical and only temporary symptoms. The revised American College of Rheumatology/European League Against Rheumatism (ACR/EULAR) Classification Criteria were established to define early RA [7]. The ACR/EULAR Classification Criteria include acute-phase reactants, serology, joint distribution, and symptom duration [8]. Erosive joint destruction, detected in conventional radiography in a typical location, is proof enough to diagnose RA. However, it is not a sign of early RA. Even during a very aggressive progression of the disease, erosive joint destruction can be identified by using conventional radiography 6–24 months at the earliest following the onset of symptoms [9, 10]. Therefore, in addition to clinical examination and serological biomarkers, imaging tools—such as ultrasound or magnetic resonance imaging (MRI)—play an important role in the detection of early RA. MRI can show changes associated with early RA that are predictive for the development of bone destruction in the course of the disease, such as soft tissue inflammation and bone edema [11, 12]. As Schleich et al. [13] have already shown, RAMRIS-5 is applicable in low-field MRI scanners. Image generation speed, spatial resolution, and contrast improve with increasing field strength. This is most noticeable between low-field (≤0.3 T) and high-field (≥1.5 T) imaging [14]. In order to detect changes associated with early RA (for example, small erosions), there is a need for a high spatial resolution and thus a high field strength. As spatial resolution increases with magnetic flux density [15–17], it can be assumed that small changes in the 3-T MRI can be detected even better than in the 1.5-T MRI. Wieners et al. [17] showed the superiority of 3-T compared with 1.5-T image quality of RA hands, regarding the extent of bone edema, synovitis, small bone erosions, and the inter-reader reliability, even though image quality at 1.5 T was also acceptable.

With the RAMRIS, the Outcome Measures in RA Clinical Trials (OMERACT) group established a highly reliable, standardized, semi-quantitative instrument to evaluate therapy outcome [13, 18, 19]. This is a sum score based on the presence of synovitis, bone marrow edema, and erosions at 23 joint sites of the dominant hand and wrist (metacarpophalangeal [MCP], intercarpal, carpo-metacarpophalangeal, radiocarpal, and radioulnar) [19]. The assessment of RAMRIS is time- and resource-consuming. A streamlined MRI score, RAMRIS-5, focusing on only five joints of the hand and wrist, has been evaluated and proven to have a strong correlation in patients with established RA at low-field MRI [13]. However, it has not yet been evaluated for patients with early RA at high-field 3-T MRI. Therefore, the aim of the study is to establish RAMRIS-5 in early RA patients at baseline and under therapy at high-field MRI within the scope of the German ArthroMark cohort, which aims to identify new therapy strategies and modern imaging for diagnosis and therapy control in early RA.

## Methods

### Patients

Twenty-eight patients with early RA (mean age: 56.8 years; minimum 39 years, maximum 74 years); rheumatoid factor (RF) or anti-cyclic citrullinated peptide (anti-CCP) antibody–positive or both; disease duration of less than 6 months, mean duration: 16.3 weeks (minimum 2 weeks, maximum 23 weeks) fulfilling the 2010 ACR/EULAR criteria for RA [8] from the German ArthroMark initiative cohort were prospectively recruited from multiple centers but MRI scans were performed in Düsseldorf only. The ArthroMark consortium—Berlin (Charité, Deutsches Rheumaforschungszentrum), Frankfurt, Munich, and Düsseldorf, Germany—was funded by the Federal Ministry of Education and Research (Bundesministerium für Bildung und Forschung [BMBF], 01EC1009).

Ethics approval was given by the ethics committee of the Heinrich-Heine University of Düsseldorf (reference number 3483) and the Charité Berlin (EA1/193/10). Written informed consent was obtained from all patients before enrollment.

All patients were treated with the recommended csDMARD, methotrexate (MTX). Supplementary application of prednisone was allowed up to 10 mg per day. No patient received a dose increase. There was no change to other treatments. With high-field MRI (3 T), imaging of the clinical dominant hand was performed at baseline (*t* = 0) before starting MTX therapy and at follow-up under MTX therapy, about 3 months (*t* = 1) and 6 months (*t* = 2) after the baseline scan. At all three examination days, the disease activity score of 28 joints (DAS-28) and C-reactive protein (CRP) were recorded.

### Magnetic resonance imaging

A 3-T MRI scanner (Magnetom Trio A Tim System; Siemens Healthcare, Erlangen, Germany) with a four-channel flex coil was used for all imaging. The image protocol contained the following sequences of the clinical dominant hand: coronal short tau inversion recovery (STIR) and T1-weighted turbo spin echo (TSE). After intravenous injection of the contrast agent (0.4 mL/kg body weight of Gd-DTPA; Magnevist^®^), a coronal TSE and a transversal SE sequence with fat suppression were applied. The field of view covered MCP II–V, carpometacarpal, carpal, radiocarpal, and distal radioulnar joints. Sequence parameters are listed in Table 1.

**Table 1:** Sequence details

Sequence/Parameter	STIR without contrast agent	T1w-TSE without contrast agent	TSE with contrast agent	SE with contrast agent
Orientation	Coronal	Coronal	Coronal	Transversal
TE/TR, ms/ms	31/5560	25/860	25/120	12/765
Flip angle, °	120	150	150	90 and 120
Slice thickness, mm	2.5	2.5	2.5	2.5
Field of view, mm × mm	120 × 120	120 × 120	120 × 120	120 × 60
Number of acquired slices	17	17	17	17

Abbreviations: *SE* spin echo, *STIR* short tau inversion recovery, *T1w-TSE* T1-weighted turbo spin echo, *TE/TR* echo time/repetition time, *TSE* turbo spin echo

### Image analyses

**Fig. 1:**
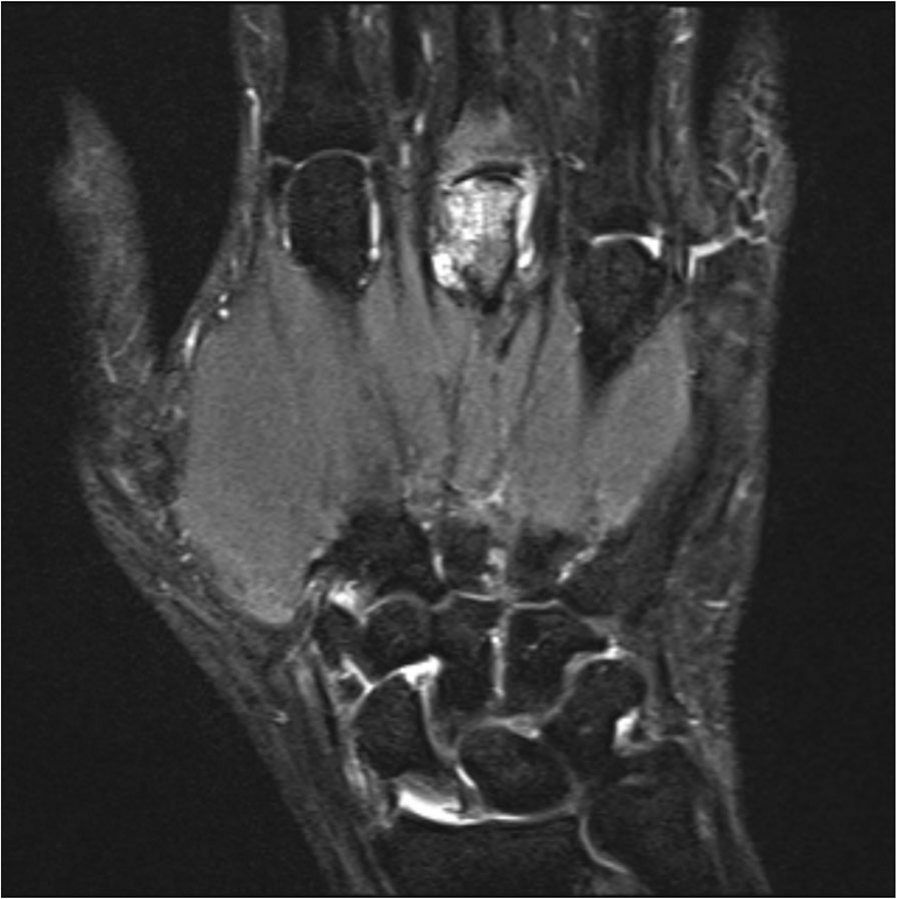
Example of a patient’s hand with bone marrow edema in the metacarpophalangeal D3 joint (coronal short tau inversion recovery without contrast agent)

**Fig. 2:**
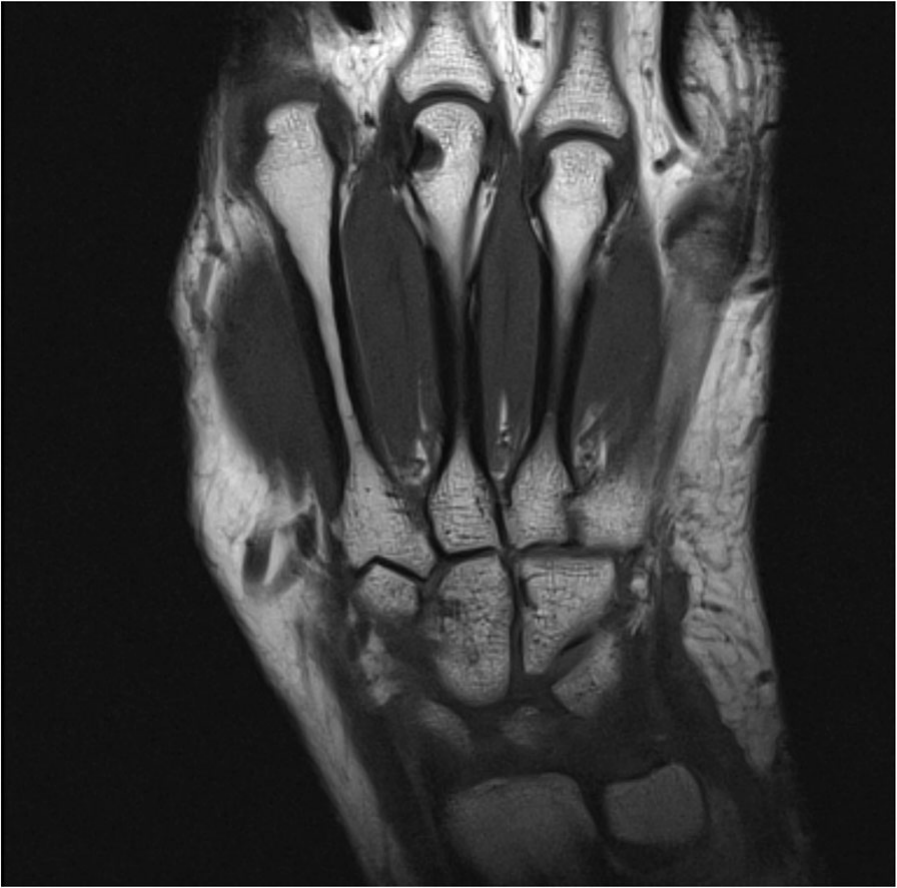
Example of a patient’s hand with erosion in the metacarpophalangeal III joint (coronal T1-weighted turbo spin echo)

**Fig. 3:**
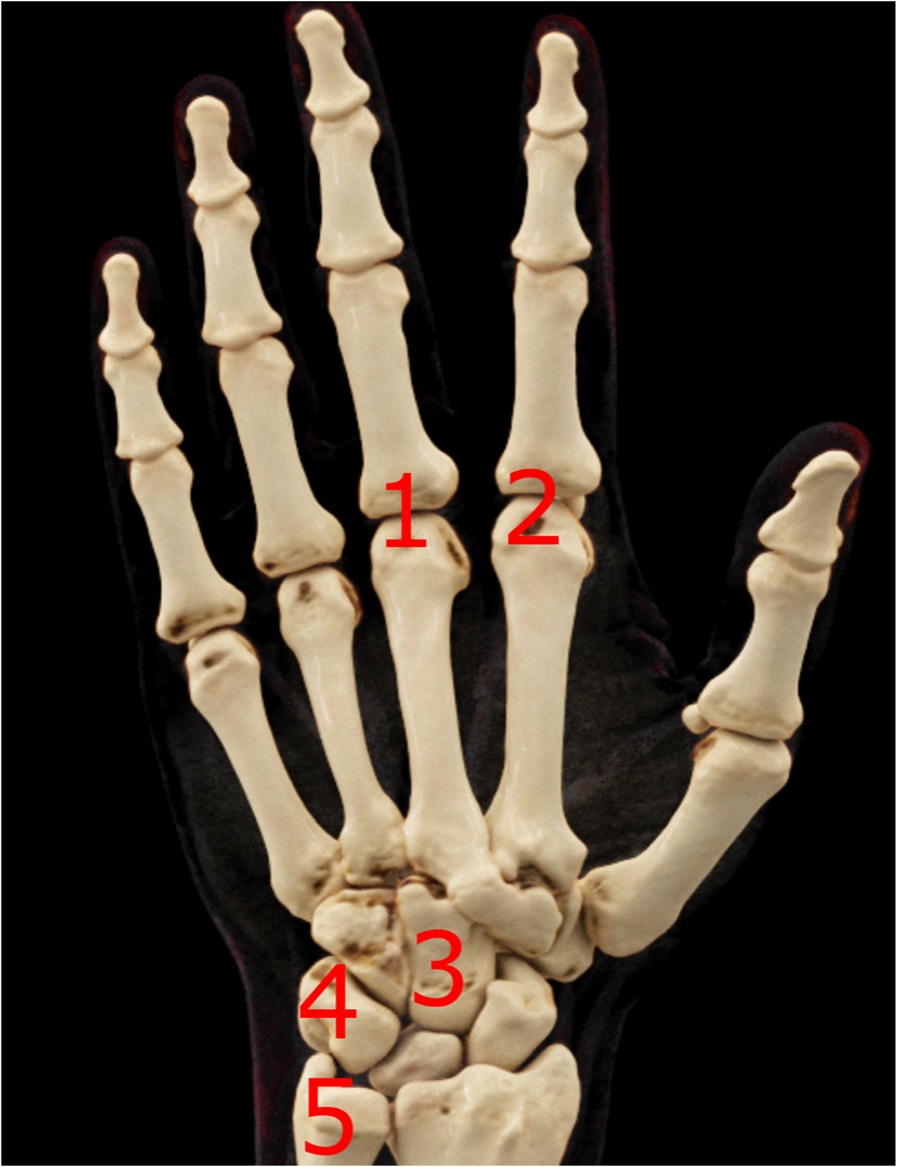
Schematic view of the five joint sites, where bone edema and erosion are evaluated for RAMRIS-5: metacarpophalangeal (MCP) III (1) and II (2) joints, capitate bone (3), triquetral bone (4), and distal ulna (5). In addition, synovitis is scored in the MCP II and III joints as well as in the wrist. Abbreviation: *RAMRIS-5* Rheumatoid Arthritis Magnetic Resonance Imaging Score for five joints of the hand

**Fig. 4:**
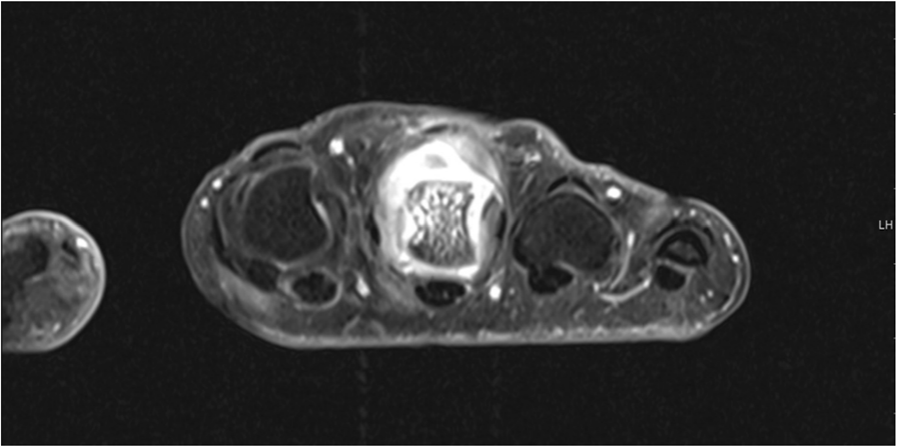
Example of a patient’s hand with synovitis metacarpophalangeal III, bone marrow edema, and erosions (transversal spin echo sequence with fat suppression)

MRI images were read in consensus by two physicians with special expertise in musculoskeletal imaging (one radiologist and one rheumatologist). MRI scans were evaluated for synovitis in the MCP joints II–V and in the wrist joints (distal radioulnar joint, radiocarpal joint, and intercarpal-carpometacarpal joints) in accordance with the EULAR/OMERACT rheumatoid arthritis MRI scoring system (RAMRIS). Additionally, bone edema and erosions were detected in MCP joint bones II–V as well as all wrist joint bones (distal radius, distal ulna, scaphoid, lunate, triquetrum, pisiform, trapezium, trapezoid, capitate, hamate, and the proximal metacarpal bones I–V) [11]. By summarizing the subscores for synovitis, bone edema, and erosions, the semi-quantitative RAMRIS score for the clinical dominant hand was calculated. RAMRIS-5, the modified, shorter version of RAMRIS, is reduced to commonly affected bones and joints in RA [13]. In order to calculate the RAMRIS-5 score, bone edema (Fig. 1) and erosion (Fig. 2) were evaluated in the following five joint sites: MCP II and III, capitate bone, triquetral bone, and distal ulna (Fig. 3). In addition, synovitis was scored in the MCP II and III joint (Fig. 4) and in the wrist. To keep it simple, there is only one synovitis wrist score that covers all intercarpal and radiocarpal joints. This simplified score corresponded to the worst synovitis score of the three affected joints.

### Statistical analysis

Standardized response means (SRMs) were calculated for all subgroups (edema, erosion, and synovitis) of RAMRIS and RAMRIS-5 after 3 months (t0 versus t1) and after 6 months (t0 versus t2). The SRM between two points in time is defined as the mean change between the two points over the standard deviation of the change between these points in time. Baseline and follow-up analyses for the total score (sum of the subscores of edema, erosion, and synovitis) were calculated in accordance with Spearman’s rank correlation coefficient. Change over time was assessed by a paired *t* test where appropriate. Results with a *P* value of less than 0.05 were considered significant. Inter-reader agreement was calculated by using Pearson’s intraclass correlation coefficient (ICC) analysis (absolute agreement). One radiologist tested the time which was used for both scoring methods.

## Results

RAMRIS-5 and RAMRIS time-comparative analysis demonstrated significantly lower time consumption for RAMRIS-5 compared with RAMRIS at baseline (42.4 ± 8.00 s versus 277.3 ± 21.3 s; *P* <0.05), at the 3-month follow-up (38.4 ± 8.70 s versus 270.6 ± 19.7 s; *P* <0.05), and at the 6-month follow-up (35.7 ± 5.70 s versus 267.2 ± 17.2 s; *P* <0.05).

RAMRIS and RAMRIS-5 were evaluated for all three subscores—bone edema, erosion, and synovitis—and for the total sum score. RAMRIS and RAMRIS-5 as a total score showed mean values at baseline (RAMRIS: 29.29; RAMRIS-5: 13.29). There was a reduction under MTX therapy already at 3-month follow-up (T0-T1 mean of differences: RAMRIS: 2.08, 95% confidence interval (CI) 0.19 to 3.98, *P* = 0.03 (paired *t* test); RAMRIS-5: 0.54, 95% CI −0.5 to 1.58, *P* = 0.29) and an increase within the 6 months of follow-up (T1-T2 RAMRIS: −2.14, 95% CI −3.61 to −0.86, *P* = 0.006, RAMRIS-5: 0.95, 95% CI −1.80 to −0.11, *P* = 0.029). The mean values for bone edema in RAMRIS and RAMRIS-5 fell continuously over time (baseline: 4.64; 1.64, 3-month follow-up: 3.21; 1.13, 6-month follow-up: 2.43; 1.04). The number of erosions increased in RAMRIS and RAMRIS-5 after 3 months and showed a further slight increase in RAMRIS-5 after 6 months (baseline: 7.96; 4.18, 3-month follow-up: 9.13; 4.92, 6-month follow-up: 9.04; 5.09). Synovitis showed a decrease after 3 months and a rise after 6 months that was below the baseline in RAMRIS and RAMRIS-5 (baseline: 16.68; 7.46, 3-month follow-up: 14.88; 6.71, 6-month follow-up: 16.26; 7.09). ICC analysis revealed a high inter-observer agreement for RAMRIS (ICC = 0.99; *P* <0.0001) and RAMRIS-5 (ICC = 0.97; *P* <0.0001).

The RAMRIS-5 total score showed a high correlation with RAMRIS at all times, at baseline and under MTX therapy (baseline: *r* = 0.838; *P* <0.001, 3-month follow-up: *r* = 0.876; *P* <0.001, 6-month follow-up: *r* = 0.897; *P* <0.001).

**Fig. 5:**
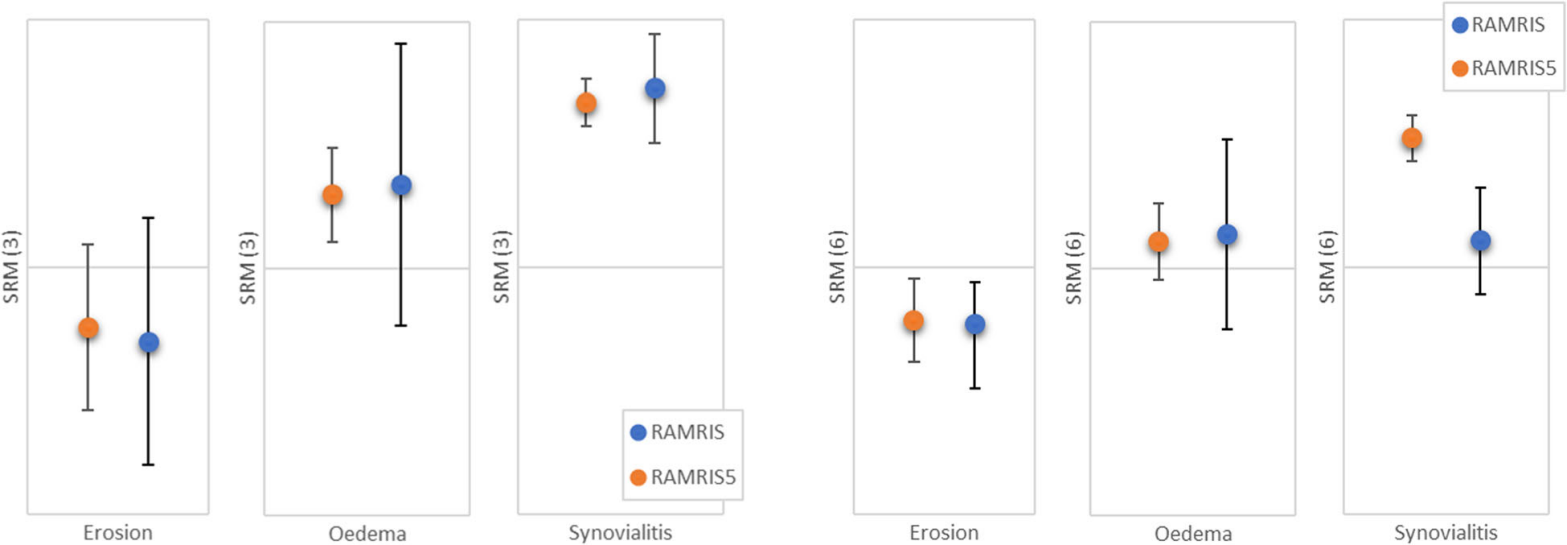
Results of the performance of detecting changes measured by the standardized response mean (SRM) for all subgroups of RAMRIS and RAMRIS-5 after 3 and 6 months of follow-up. Abbreviations: *RAMRIS* Rheumatoid Arthritis Magnetic Resonance Imaging Score, *RAMRIS-5* Rheumatoid Arthritis Magnetic Resonance Imaging Score for five joints of the hand

In the short term (3-month follow-up), RAMRIS and RAMRIS-5 showed a similar ability for detecting changes with overlapping standard deviations for all subgroups (bone edema, erosion, and synovitis). In the long-term comparison (6-month follow-up), the RAMRIS-5 also showed similar capabilities to detect changes regarding bone edema and erosion compared with RAMRIS. Deviations occurred only regarding synovitis, where the change is slightly higher in RAMRIS-5 (standardized response mean SRM(R) = 0.07 ± 0.14; SRM(R5) = 0.34 ± 0.06) (Table 2, Fig. 5).

**Table 2:** Comparison of standardized response means for the subgroups erosion, edema, and synovitis for months 3 and 6

	SRM (3)	SD (3)	SRM (6)	SD (6)
Erosion				
RAMRIS	−0.15	0.25	−0.15	0.11
RAMRIS-5	−0.12	0.17	−0.14	0.17
Edema				
RAMRIS	0.17	0.29	0.09	0.25
RAMRIS-5	0.15	0.9	0.07	0.10
Synovialitis				
RAMRIS	0.47	0.14	0.07	0.14
RAMRIS-5	0.43	0.06	0.34	0.06

*Abbreviations*: *RAMRIS* Rheumatoid Arthritis Magnetic Resonance Imaging Score, *RAMRIS-5* Rheumatoid Arthritis Magnetic Resonance Imaging Score for five joints of the hand, *SD* standard deviation, *SRM* standardized response mean

CRP levels were highest at the initial measurement (9.6 mg/L) and continually decreased in the 3 and 6 months of follow-up under MTX therapy (6.5 mg/L; 3.6 mg/L). In 14 out of 24 patients, CRP levels decreased in the 3-month follow-up after MTX therapy. There were five patients with constant CRP levels and five patients with an increase. In the 6-month follow-up in 16 out of 22 patients, CRP levels decreased. There were five patients with no changes in CRP levels and one with an increase.

Corresponding to CRP levels, the DAS-28 had been highest at the initial measurement (4.69) and continually dropped in the 3 and 6 months of follow-up (3.46; 2.57). In 21 out of 24 patients, DAS-28 improved by the 3-month follow-up. There were three patients with a DAS-28 increase. By the 6-month follow-up, DAS-28 decreased in 22 out of 23 patients. There was one patient with an increase (Table 3).

**Table 3:** Mean clinical and radiological measures before and after therapy (3- and 6-month follow-up)

	DAS	CRP	RAMRIS				RAMRIS-5			
			Edema	Synovitis	Erosion	RAMRIS	Edema	Synovitis	Erosion	RAMRIS-5
t0	4.69	0.96	4.64	16.68	7.96	29.29	1.64	7.46	4.18	13.29
t1	3.46	0.65	3.21	14.88	9.13	23.32	1.13	6.71	4.92	10.93
t2	2.57	0.36	2.43	16.26	9.04	22.79	1.04	7.09	5.09	10.86

*Abbreviations*: *CRP* C-reactive protein, *DAS* disease activity score, *RAMRIS* Rheumatoid Arthritis Magnetic Resonance Imaging Score, *RAMRIS-5* Rheumatoid Arthritis Magnetic Resonance Imaging Score for five joints of the hand

Furthermore, there was a weak correlation between 6-month RAMRIS follow-up and DAS-28 at the 3-month follow-up (*r* = 0.533; *P* = 0.013). We did not find evidence for a correlation between RAMRIS/RAMRIS-5 and DAS-28 or CRP (*t* = 0: DAS-28/RAMRIS *P* = 0.657; DAS-28/RAMRIS-5 *P* = 0.888; CRP/RAMRIS *P* = 0.267; CRP/RAMRIS-5 *P* = 0.303; *t* = 1: DAS-28/RAMRIS *P* = 0.055; DAS-28/RAMRIS-5 *r* = 0.434; *P* = 0.034; CRP/RAMRIS *P* = 0.127; CRP/RAMRIS-5 *r* = 0.496; *p* = 0.14; *t* = 2: DAS-28/RAMRIS *P* = 0.629; DAS-28/RAMRIS-5 *P* = 0.543; CRP/RAMRIS *p* = 0.731; CRP/RAMRIS-5 *P* = 0.816).

## Discussion

The OMERACT RAMRIS system is widely accepted as a reference standard in RA trials for diagnosing, staging, and follow-up [20]. Owing to its time commitment, it is barely used in clinical practice [20]. A simplified scoring system, RAMRIS-5, introduced by Schleich et al., turned out to be a time-saving alternative with close correlation to RAMRIS for patients with an established RA in low-field MRI (minimum disease duration of 5 years) [13]. Because it was unclear whether there is also a high correlation between RAMRIS and RAMRIS-5 in patients with early RA, we evaluated the shortened scoring method RAMRIS-5, reduced to only five instead of 23 joint sites, for patients earlier than 6 months after disease onset (early RA).

The selection of RAMRIS-5 joints is based on previous studies and observations of our own research group [18, 21] which demonstrated a frequent involvement of the selected joints. With regard to early RA, Fleming et al. [22] describe lesions at the MCP joints as well as at the wrist, which are partly included in the RAMRIS-5. The RAMRIS-5 therefore seemed to be suitable primarily for the early stage of RA.

Our results show a strong correlation between the total mean RAMRIS and the total mean RAMRIS-5 at baseline as well as under MTX therapy at 3 and 6 months of follow-up. This emphasizes that RAMRIS-5 is an appropriate, time-saving alternative to RAMRIS not only for patients with established RA but also for patients with early RA. It is suitable for detecting disease-typical findings and follow-up evaluation under therapy.

Furthermore, RAMRIS-5 has an equivalent performance level for detecting changes under therapy as RAMRIS does for all subgroups (edema, erosion, and synovitis) after 3 months. Even after 6 months, change is also similar between RAMRIS-5 and the original score for edema and erosion. Deviations between RAMRIS and RAMRIS-5 occurred only in the change of synovitis in the long follow-up (6 months). The change of the RAMRIS-5 synovitis score was higher than that of RAMRIS. In our case, this means a stronger reduction of RAMRIS-5 than of RAMRIS after therapy.

The fact that synovitis is measured in only three instead of five regions, in contrast to the other subgroups, increases the risk of deviating from the results of the original score. In addition, the wrist score for synovitis was deliberately chosen as a region frequently affected by RA. In case of a decrease in synovitis under therapy, this leads to a stronger weighting of the improvement in RAMRIS-5 and a discrete overestimation of the improvement in the course of the disease. One might argue to refine the RAMRIS-5 synovitis score by including another joint area, but because the synovial change is overestimated and not underestimated, the RAMRIS-5 does not miss out on a possible therapy-requiring disease relapse. On the contrary, progress in synovitis could be even better perceived. Furthermore, the change in the “window of opportunity” after about 3 months is similar to that of the original RAMRIS and deviates from it only in the later course (6-month follow-up). Furthermore, since the great advantage of RAMRIS-5 is its brevity and suitability for everyday use, adding more joint sites is not recommended from our point of view for implementing MRI in daily practice. To summarize, we are convinced that the selected joints in RAMRIS-5 are still the right choice for the assessment of early RA disease activity at the beginning and during treatment, even for early RA. We could show the time-saving capabilities of RAMRIS-5, which is a further and very important step to implement an objective MRI scoring method in clinical routines.

Early diagnosis and therapy of RA are of great importance to prevent joint destruction and to reach the stated target of remission or at least low disease activity [5, 6]. Conventional x-rays of the hand and wrist are still the gold standard for diagnosing, staging, and follow-up of patients with RA. However, it is insensitive to early erosion whereas MRI is more sensitive for detecting erosions and other early changes associated to RA like bone edema and synovitis [23, 24]. Therefore, MRI has become a useful tool in the diagnostic process for arthritis, especially for early RA [17]. Regarding MRI image quality, there is a better signal-to-noise ratio and a higher spatial resolution with increasing magnetic flux density in general [15–17]. Additionally, compared with the image quality of low-field MRI (<0.3 T), that of high-field MRI (≥1.5 T) is superior because of a reduced acquisition time that leads to shorter protocols and fewer motion artifacts [15, 25]. Other technically related non-RA-specific studies showed that, compared with the image quality of low-field MRI, that of high-field MRI systems is superior when focusing on small anatomic structures, such as the posterior inter-malleolar ligament (a possible cause of the posterior ankle impingement syndrome) [16]. Therefore, it can be assumed that RA-specific small anatomical structures can also be better detected by means of high-field MRI. Moreover, there is evidence that 3-T is superior to 1.5-T MRI for the detection for bony changes [26]. Owing to the higher spatial resolution at 3 T, bone marrow edema was better assigned to anatomic structures [17], which might help to diagnose early RA. Even though some studies declare low-field imaging to be a good alternative [17], there is no doubt that detailed high-field MRI offers a better image quality compared with low-field imaging [17]. In summary, it can be stated that the anatomically small changes in early RA are better represented by the better spatial resolution and the higher contrast in high-field MRI. If 3-T MRI is available, the higher field strength should be preferred.

As expected, levels of CRP, DAS-28, RAMRIS, and RAMRIS-5 initially showed maximum value and dropped in the 3 months of follow-up under MTX therapy, indicating response to the therapy. Moreover, it confirms RAMRIS/RAMRIS-5 as good monitoring tools. Surprisingly, even though CRP and DAS-28 dropped in the 6 months of follow-up, there was a slight increase in RAMRIS/RAMRIS-5. Consequently (and consistent to the results of Schleich et al.), there was no significant correlation between RAMRIS/RAMRIS-5 and DAS-28 or CRP. Some authors interpret the missing correlation as a result of MRI superiority in detecting RA-associated inflammation compared with clinical assessment or serological parameters [13]. Indeed, it is known that MRI is very sensitive for the detection of even very small pathologies [24]. In fact, Sewerin et al. documented a progression in erosive bone destruction detected by MRI and an increase in the RAMRIS during improvement of DAS-28 or EULAR remission for up to 40% of patients [27]. The missing correlation between RAMRIS/RAMRIS-5 and DAS-28/CRP could demonstrate that there may be a progression of local synovial destructive reaction that is visible by MRI despite clinical response or even remission. In other words, the missing correlation could be a sign of silent progression [18, 28]. It must be mentioned that the very high sensitivity could lead to overinterpretation of MRI-detected pathologies. Hence, several studies could demonstrate high numbers of erosions, even in healthy controls. Boeters et al. recently demonstrated that MRI-detected erosions have to be assessed very carefully, as erosion scores of individual persons with and without RA were largely overlapping, and even RA-specific erosions were found in both groups. This underlines the need for re-evaluating the comparability of the RAMRIS and RAMRIS-5 in high-field MRI [29].

Our study had several limitations. We had a homogenous patient cohort but a fairly small patient number. This is partly a result of the study’s strict requirement to include only patients with early RA who were investigated three times. Larger patient cohorts are needed to prove whether RAMRIS-5 is a valid and reliable alternative to the time-consuming RAMRIS for all patients. If it is not, outliers must be identified. It is also necessary to perform further studies to confirm and investigate the lack of correlation between RAMRIS/RAMRIS-5 and CRP/DAS-28 and to show whether a silent progression might be a persuasive explanation.

In summary, this study underlines the former data showing a very high correlation between RAMRIS and RAMRIS-5. It was not yet known that RAMRIS-5 is verified as a good and resource-saving tool for diagnosing and follow-up investigations even in early RA when using high-field (3-T) MRI. RAMRIS-5 presents itself as a score that detects similar changes as the original score with only a slightly higher detection of change in the synovitis subscore. Despite the limitations that a reduced score always has, the RAMRIS-5 shows very good results and is a useful tool in clinical, everyday life because of its great time efficiency.

## Conclusion

RAMRIS-5, the simplified version of the well-established RAMRIS, is a resource-saving, appropriate alternative with an accurate detection of change over time, especially for edema and erosion. It is appropriate not only for patients with established RA but also for those with early RA. In regard to its shorter expenditure of time, there may be a high potential for using RAMRIS-5 in daily clinical practice to detect and monitor RA.

## Abbreviations

ACR: American College of Rheumatology
CI: Confidence interval
CRP: C-reactive protein
csDMARD: Conventional synthetic disease-modifying anti-rheumatic drug
DAS-28: Disease activity score of 28 joints
EULAR: European League Against Rheumatism
ICC: Intraclass correlation coefficient
MCP: Metacarpophalangeal
MRI: Magnetic resonance imaging
MTX: Methotrexate
OMERACT: Outcome Measures in Rheumatoid Arthritis Clinical Trials
RA: Rheumatoid arthritis
RAMRIS: Rheumatoid Arthritis Magnetic Resonance Imaging Score
RAMRIS-5: Rheumatoid Arthritis Magnetic Resonance Imaging Score for five joints of the hand
SRM: Standardized response mean
TSE: Turbo spin echo
